# Magnetic State Generation using Hamiltonian Guided Variational Autoencoder with Spin Structure Stabilization

**DOI:** 10.1002/advs.202004795

**Published:** 2021-03-24

**Authors:** Hee Young Kwon, Han Gyu Yoon, Sung Min Park, Doo Bong Lee, Jun Woo Choi, Changyeon Won

**Affiliations:** ^1^ Center for Spintronics Korea Institute of Science and Technology Seoul 02792 South Korea; ^2^ Department of Physics Kyung Hee University Seoul 02447 South Korea

**Keywords:** energy minimization, generative model, machine learning, micromagnetism, the ground state

## Abstract

Numerical generation of physical states is essential to all scientific research fields. The role of a numerical generator is not limited to understanding experimental results; it can also be employed to predict or investigate characteristics of uncharted systems. A variational autoencoder model is devised and applied to a magnetic system to generate energetically stable magnetic states with low local deformation. The spin structure stabilization is made possible by taking the explicit magnetic Hamiltonian into account to minimize energy in the training process. A significant advantage of the model is that the generator can create a long‐range ordered ground state of spin configuration by increasing the role of stabilization even if the ground states are not necessarily included in the training process. It is expected that the proposed Hamiltonian‐guided generative model can bring about great advances in numerical approaches used in various scientific research fields.

## Graphical abstract

An energy‐minimization variational autoencoder model is devised to generate energetically stable physical states by taking the explicit Hamiltonian into the training process. A significant advantage of the model is that the generator can produce the ground states of various systems even if the ground states are not necessarily included in the training process.

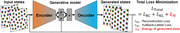

## 1 Introduction

Computational approaches have been extensively used across all scientific research fields in order to investigate the unexplored nature of numerous systems. In material science and condensed matter physics, numerical simulation of physical states, such as the Monte Carlo method, allows for a better understanding of experimental results. Recently, machine learning technique has emerged as a new computational approach in a wide range of disciplines. In condensed matter physics, it has been employed to solve strongly coupled many‐body problems,^[^ ^1^, ^2^ ^]^ to simulate the ground states of various systems,^[^ ^3^, ^4^ ^]^ and to estimate Hamiltonian parameters from experimentally observations.^[^ ^5^, ^6^, ^7^ ^]^

Numerical methods are also extensively utilized in magnetism research. Unique magnetic states such as magnetic skyrmions ^[^ ^8^, ^9^, ^10^, ^11^, ^12^, ^13^, ^14^ ^]^ and spiral structures ^[^ ^15^, ^16^, ^17^, ^18^, ^19^ ^]^ are intensively studied using micromagnetic simulations to investigate their stabilities, generation conditions, and dynamic properties; understanding the characteristics of these microscopic magnetic states is a key prerequisite for developing novel spintronic devices. In line with its widespread usage in other fields, the aforementioned machine learning models are being adopted and developed to explore classical and quantum spin states,^[^ ^20^, ^21^ ^]^ to increase the resolution of spin configuration images shown on a square grid system,^[^ ^22^ ^]^ to investigate the phase transition of magnetic systems,^[^ ^23^, ^24^ ^]^ and to implement plausible temperature fluctuations.^[^ ^25^, ^26^ ^]^

Generating new data from a given data set is one of the areas in which the machine learning techniques can be most effectively applied. Indeed, several generative network models, such as variational autoencoder (VAE)^[^ ^27^ ^]^ and generative adversarial network,^[^ ^28^ ^]^ have been devised for that purpose. Unlike traditional numerical methods, such as simulated annealing, these generative models can yield numerous new data immediately after the training process. This advantage will not only aid in statistical analysis on various physical states but will also be used to investigate new physical states. However, the states produced by these generative models may have incorrect physical properties due to various extrinsic factors (e.g., numerical artifacts, injected local noises, and blurring effects).^[^ ^29^ ^]^

In this study, we propose a VAE model in which the magnetic Hamiltonian calculation explicitly participates in the training process. We find that this VAE model, referred to as energy‐minimization VAE (E‐VAE), can be effectively utilized to create magnetic states which possess increased stability. Using magnetic skyrmion configurations implemented by the 2D Heisenberg spin system as the input data, two key properties are evaluated to demonstrate the advantage of the E‐VAE model compared to the standard VAE. One is the capability of generating various spin configurations different from, yet resembling, the input data, i.e., the model should be generative. The other is the stability of the output spin configurations, i.e., the results from the model should be physically reasonable. Detailed analysis is conducted to examine the effects of energy minimization on both the generated spin configurations and the latent space—a multidimensional space containing the compressed representations of the input data. We find that the latent space is modulated in the E‐VAE model, which includes the magnetic Hamiltonian, so that energetically stable states can be sampled more frequently in the generation processes, resulting in more physically plausible output data. Additionally, a critical change occurs in the behavior of E‐VAE as the influence of Hamiltonian increases. After the critical change, the energy minimization term dominates such that the generated spin configurations are in the ground state of the system. This unique feature, i.e., efficient searching of the ground spin configuration, demonstrates the power and potential of our method.

## 2 Strategy

### 2.1 Test System and Dataset Generation

A dataset should be chosen to best evaluate the main purpose of VAE, which is the production of plausible data. The input data should be rich in structure, yet have inclusive properties suitable for quantitative evaluation. For that purpose, the magnetic skyrmion configurations formed on a 2D magnetic system are used as the dataset in this study. Magnetic skyrmions have been intensively studied in numerous numerical and experimental investigations^[^ ^8^, ^9^, ^10^, ^11^, ^12^, ^13^, ^14^, ^30^, ^31^, ^32^, ^33^ ^]^ owing to its intriguing fundamental physical characteristics and potential technological application possibilities. The abundance of well‐understood characteristics of magnetic skyrmions, such as its structural form and topological properties, provides advantages in evaluating whether a trained VAE can generate physically plausible states.

Magnetic skyrmion configurations are formed and stabilized by the competition between the exchange interaction and Dzyaloshinskii–Moriya (DM)^[^ ^34^, ^35^ ^]^ interaction under an external field applied along the out‐of‐plane direction of a 2D system. We use a square grid lattice system (128  ×  128 size) with the Heisenberg spin model to implement the 2D magnetic systems. The simple Hamiltonian model, H, used to generate the magnetic skyrmion configurations is shown in Equation (1)

(1) $$H\;=\;-J\sum_{\left\langle i,j\right\rangle }{\vec{S}}_{i}\cdot {\vec{S}}_{j}-\sum_{\left\langle i,j\right\rangle }{\vec{D}}_{ij}\cdot \left({\vec{S}}_{i}\times {\vec{S}}_{j}\right)-{\vec{h}}_{\mathrm{ext},z}\cdot \sum_{i}{\vec{S}}_{i}$$

where ${\vec{S}}_{i}$ is a normalized spin vector with three components, *S_x_*, *S_y_*, and *S_z_* on *i*th grid site, *J* is the exchange interaction strength, ${\vec{D}}_{ij}$ is the DM vector for the DM interaction between *i* and *j* grid sites, and ${\vec{h}}_{\mathrm{ext},z}$ is the applied out‐of‐plane magnetic field.

The ground state skyrmion configuration has long‐range order: the skyrmions form a triangular lattice, which is known as a skyrmion lattice.^[^ ^13^, ^14^, ^33^ ^]^ The energy minimization conditions for this ground state are well investigated. However, conventional numerical methods, such as a simulated annealing process, does not guarantee the generation of fully ordered skyrmion lattice structures; since the Hamiltonian in Equation (1) is composed of short‐range interactions, domains are formed locally in the annealing process, such that an energy barrier needs to be overcome for these domains become oriented. Hence, it is challenging to numerically generate an ordered ground state skyrmion configuration.

We generate a total dataset composed of ≈40 000 skyrmion configurations using a simulated annealing process implemented by the Monte Carlo method.^[^ ^13^, ^33^, ^36^ ^]^ In order to focus on the investigation on the characteristics of E‐VAE in this study, the magnitudes of Hamiltonian parameters are fixed at *J*  =  1, $|{\vec{D}}_{ij}|$ = 0.3, and $|{\vec{h}}_{\mathrm{ext},z}|$ = 0.05 which are appropriate values to form magnetic skyrmions in a 2D magnetic system.^[^ ^13^, ^33^ ^]^ Detailed information about the data generation is given in the Experimental Section.

### 2.2 Network Structure

**Figure 1:**
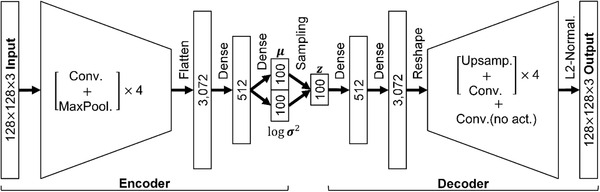
Variational autoencoder network structure. A schematic diagram of the VAE network structure used in this study. The “Conv.,” “Dense,” “MaxPool.,” and “Upsamp.” indicate convolutional neural network layer, fully connected neural network, max‐pooling layer, and upsampling layer, respectively. See the Experimental Section for a detailed explanation.

We construct a network structure as shown in **Figure** **1**, which is similar to VAE network structures used in previous studies.^[^ ^27^, ^37^ ^]^ First, the input spin configuration, *X*_In_, is encoded into a combination of the mean (*μ*) and log‐variance (log *σ* ^2^) values through the encoder network; 100 values are encoded for each mean and log‐variance in this study. Second, a set of values, *z*, is sampled from a set of normal distributions, *P*(*z*|*X*_In_), which are modulated using the encoded *μ* and *σ* values. This *z* is considered as a code or a vector composed of the underlying feature values of the data, and referred to herein as the sampled latent vector. Lastly, *z* is decoded into an output spin configuration, *X*_Out_, through the decoder network. Detailed information about the network structures is given in the Experimental Section.

In addition to obtaining *X*_Out_ through an input‐dependent generation process, another general purpose of the generative models is to produce output results without input data, using only the features understood during the training process. For the generation process which is input‐independent, the sampled latent vector can be produced from a standard normal distribution, *P*(*z*), which does not require the encoded *μ* and *σ* values. In this case, only the decoder network is used to generate the output, and the output spin configuration from *P*(*z*) is called herein as *X*'_Out_.

A normalization process, L2‐normalization, is added at the last part of our decoder, in contrast to typical VAE network structures. The necessity of the process owes to the fact that all the spins, $\vec{S}$s, in the Heisenberg spin model are unit vectors. The process is indicated by $L2-\mathrm{normal}.\;=\vec{S}/\sqrt{\sum_{n=x,y,z}{S_{n}}^{2}}\;$in Figure 1.

### 2.3 Total Loss Function of E‐VAE

E‐VAE is trained to minimize the total loss, *L*_Total_, which is composed of three loss terms as in Equation (2), with each loss term expressed in Equation (3).

(2) $$L_{\mathrm{Total}}=L_{\mathrm{RC}}\;+\beta L_{\mathrm{KL}}+\gamma L_{H}$$

(3) $$\begin{array}{c} \;L_{\mathrm{RC}}={\langle {\left(S_{\mathrm{In},i,\alpha }-S_{\mathrm{Out},i,\alpha }\right)}^{2}\rangle }_{i,\alpha \left(=x,y,z\right)}\;\\ \;L_{\mathrm{KL}}=\frac{1}{2}\;\sum_{n\;=\;1}^{100}\left(\sigma_{n}^{2}+\mu_{n}^{2}-\ln\left(\sigma_{n}^{2}\right)-1\right)\\ \;L_{H}=\frac{H_{\mathrm{Out}}-E_{0}}{N}\\ \;=\frac{1}{N}\;\left(-E_{0}-J\sum_{\langle i,j\rangle }{\vec{S}}_{\mathrm{Out},i}\cdot {\vec{S}}_{\mathrm{Out},j}-\sum_{\langle i,j\rangle }{\vec{D}}_{ij}\cdot \left({\vec{S}}_{\mathrm{Out},i}\times {\vec{S}}_{\mathrm{Out},j}\right)-{\vec{h}}_{\mathrm{ext},z}\cdot \sum_{i}{\vec{S}}_{\mathrm{Out},i}\right) \end{array}$$

The first term in Equation (3), *L*_RC_, is the reconstruction loss to make *X*_Out_ identical to *X*_In_. The *S*_In,*i*,*α*_ and *S*_Out,*i*,*α*_ indicate the *α* (  = *x*, *y*, *z* ) components of the spins located at *i*th grid site in *X*_In_ and *X*_Out_, respectively. The second term, *L*_KL_, is the Kullback–Leibler (KL) loss measuring the similarity between a standard normal distribution and target distribution;^[^ ^27^, ^37^ ^]^ in usual VAE studies, the target distribution is a Gaussian distribution with the mean and standard deviation values encoded from the input data. The goal of the minimization of *L*_KL_ term is to make all the *μ*_*n*_ and *σ*_*n*_ to be 0 and 1 respectively, where the *μ*_*n*_ and *σ*_*n*_ are the mean and standard deviation values encoded from *X*_In_ using the encoder network structure shown in Figure 1. The last term, *L*_H_, is the Hamiltonian loss term first proposed in this study and not included in the standard VAE. It prefers the energy of *X*_Out_ to be minimized. *E*_0_ is − 2*JN* which is the energy of a uniformly magnetized state (*N* is the total number of spins), such that $-E_{0}-J\sum_{\left\langle i,j\right\rangle }{\vec{S}}_{\mathrm{Out},i}\cdot {\vec{S}}_{\mathrm{Out},j}$ can be considered to be the exchange energy cost for the formation of multidomain magnetic structures in *X*_Out_. *β* and *γ* are the controlling parameters for the independent variation of the magnitudes of *L*_KL_ and *L*_H_, which enables investigating the effect of each loss term.

During the training process, these loss terms are under competition with one another. The *L*_RC_ term induces our network structure to learn a representation of the input data by reducing the dimensions of information. In order for the output result to accurately reconstruct the input data, the acquired representation should be composed of various numbers containing characteristics of the input data. On the other hand, the minimization of the *L*_KL_ term prefers that both *μ*_*n*_ and *σ*_*n*_ to be simple constants, 0 and 1, independent of the input data. Thus, the *L*_KL_ term competes with *L*_RC_, which allows the trained network to generate various spin configurations that are not identical to *X*_In_s. The minimization of *L*_H_ prevents the formation of unstable local structures among *X*_Out_s, so that the output structure is physically reasonable. Note that the *X*_In_s used in this study are metastable states composed of locally stable magnetic structures. Since the *X*_In_s are not in the ground states but in metastable states, the emphasis on *L*_H_ makes the energy further lowered from *X*_In_s, which is against the tendency of *L*_RC_. Therefore, *L*_H_ has complex relationships with both the *L*_RC_ and *L*_KL_ terms.

## 3 Result

### 3.1 Training Results of Standard VAE

We first investigate the characteristics of standard VAE without the Hamiltonian loss (*γ*  =  0), i.e., the Hamiltonian loss term *L*_H_ is not included in this standard model. We increase *β* from a small value (10^−6^) to 1 sequentially to search a proper value of *β* which can make a trained VAE generate plausible spin configurations. The details of this sequential training process are described in the Experimental Section. The *X*_Out_s for several *β* cases are given in Figure S1 in the Supporting Information to support the discussions below.

**Figure 2:**
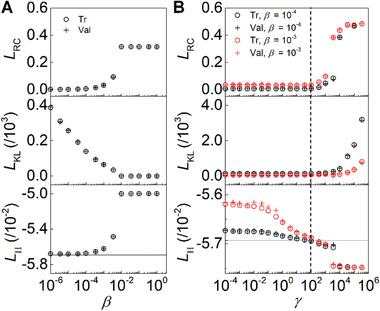
Losses of trained VAE and E‐VAE. Loss values calculated using training (Tr) and validation (Val) dataset during the training processes of A) standard VAE and B) E‐VAE. The loss values shown in the graphs are calculated after every 100 training epochs completes in one sequential training process discussed in the Experimental Section. The solid lines in the graphs of *L*_H_ in (A) and (B) are the averaged energy values of spin configurations in our training dataset. The dashed line in (B) shows the boundary of two distinct regions with different characteristics of *L*_RC_.

**Figure** **2A** shows the behavior of each loss term during the training progress. When *β* is small (10^−6^ −10^−4^), *L*_RC_ is almost zero, indicating that the VAE replicates *X*_Out_s from *X*_In_s. As *β* increases up to 10^−2^, *L*_RC_ increases exponentially and *L*_KL_ decreases gradually. At *β* ≈ 10^−2^, the reconstruction loss *L*_RC_ is large, implying that the trained VAE begins to generate various spin configurations which are not necessarily identical to *X*_In_. We plot *L*_H_ (last row of Figure 2A) to investigate the energy of the generated spin configurations, although *L*_H_ is not included in the training process of the standard VAE. *L*_H_ shows a similar behavior with *L*_RC_. Since the *X*_Out_s generated in the low *β* (10^−6^ −10^−4^) are nearly identical to the *X*_In_s, the energy of the *X*_Out_s is also nearly identical to those of the *X*_In_s. Increasing *β* causes *L*_H_ to increase as well, indicating that the resulting spin configuration becomes less energetically stable. Therefore, the standard VAE may provide richness to the output structure, but fails to maintain energetic stability.

The behavior of the standard VAE shows a critical transition at *β* ≈ 10^−2^. If *β* exceeds this critical value, the role of *L*_KL_ dominates as the other loss term *L*_RC_ becomes constant. This phenomenon, referred to as a posterior‐collapse (PC) in the VAE research field,^[^ ^37^, ^38^ ^]^ indicates a failure in encoding meaningful information from the input data, resulting in the generation of only trivial output data. In our study, the spin configurations generated after the occurrence of PC is an out‐of‐plane single domain where all the spins are aligned uniformly (see Figure S1, Supporting Information). The increasing *L*_H_ in Figure 2A clearly shows that the resulting out‐of‐plane single domain is not an appropriate ground state.

### 3.2 Characteristics of E‐VAE

The results from the standard VAE confirm that the proper range of *β* is in the range from 10^−4^ to 10^−2^ in which the VAE can produce various structures while PC does not occur. We choose two *β*s within the range to investigate how the E‐VAE model enhances the capabilities of the VAE. With the *β* values fixed at either 10^−4^ or 10^−3^, we additionally consider the effect of *L*_H_, sequentially increasing *γ* value from 10^−4^ to nearly 10^6^. The details of the sequential training process are discussed in the Experimental Section.

The results from the E‐VAE in Figure 2B clearly demonstrate that including *L*_H_ improves the performance of the VAE in several aspects. First, we notice that it enhances the stability of the generated *X*_Out_s. During the training process, *L*_H_ are reduced as *γ* increases (lower panel in Figure 2B), indicating that the energy minimization of *X*_Out_s has been successfully performed. Second, these graphs are divided into two distinct regions showing different behaviors of *L*_RC_ depending on the range of *γ* (the two regions are divided by the dashed line in Figure 2B): *L*_RC_ hardly changes at *γ* ≤ 10^2^, but dramatically increases at *γ* > 10^2^. Through the distinct behaviors of the *L*_RC_, we infer that the role of *L*_H_ in the training process is altered at *γ* ≈ 10^2^. When *γ* is small, we speculate that small‐scale modifications on the *X*_Out_s, such as a reduction of local noises, occur to minimize local energy. These small‐scale differences are not noticeable since *L*_RC_ is the squared differences between the input and output as shown in Equation (3). When *γ* is large, the dramatic increase of the *L*_RC_ term implies that the difference between input and output becomes larger. In this case, we speculate that the trained E‐VAE can generate *X*_Out_s which are completely different spin configurations from the *X*_In_s. Moreover, the output *X*_Out_s are in the lower energy states than the input *X*_in_s. Note that the solid line in the graph for *L*_H_ in Figure 2B indicates the average energy value of spin configurations in the training dataset. The last important observation in Figure 2B is the presence of a critical transition at very large *γ*: *L*_H_ drops to significantly lower energy values at *γ* ≈ 10^3^ ≈ 10^4^ for both *β* values (see Section 3.5 for more discussion on this sudden drop in the energy loss term). In this region, *L*_RC_ is saturated and *L*_KL_ increases exponentially.

### 3.3 Local Noise Reduction

**Figure 3:**
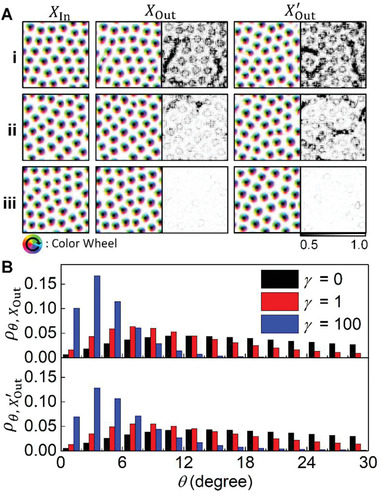
Sampled spin configurations and local energy distributions. A) Several input and output spin configurations fed to and generated from the generative models trained under i) *γ*  =  0 (= standard VAE), ii) *γ*  =  1, and iii) *γ*  =  100 conditions. The color wheel and black/white contrast indicate the in‐plane and out‐of‐plane magnetization directions, respectively. The grayscale maps, on the right side of *X*_Out_ and $X_{\mathrm{Out}}^{\prime }$ panels, show the spatial distributions of the local correlation between the spins and effective fields. B) The normalized density functions showing the frequency of $\theta \;=\cos^{-1}\left(\vec{S}\cdot {\vec{h}}_{\mathrm{eff}}/|\vec{S}\left|\right|{\vec{h}}_{\mathrm{eff}}|\right)\;$in the generated spin configurations. The $\rho_{\theta ,X_{\mathrm{Out}}}$ is calculated using all *X*_Out_s generated when the total spin configurations in the test dataset are fed into each trained model and the $\rho_{\theta ,X_{\mathrm{Out}}^{\prime }}$ is calculated using a number of $X_{\mathrm{Out}}^{\prime }$s equal to the number of spin configurations in the test dataset.

In order to understand how the stability is improved in the output results generated through E‐VAE in the small *γ* region (*γ* ≤ 10^2^), we randomly select several input and output spin configurations and visualize the local correlation maps between the spins and the effective fields, ${\vec{h}}_{\mathrm{eff}}$ ($=\;-\frac{\partial H}{\partial \vec{S}}$) in **Figure** **3A**. The local correlation is $\cos\left(\theta \right)=\vec{S}\cdot {\vec{h}}_{\mathrm{eff}}/|\vec{S}\left|\right|{\vec{h}}_{\mathrm{eff}}|\;$where *θ* represents the angles between the spins and the effective fields; this provides a measure of the stability since each spin has the lowest energy when it is parallel to the effective field. Comparing the images shown in Figure 3A, we confirm that the consideration of Hamiltonian loss reduces the unstable local irregular structures in both *X*_Out_s and $X_{\mathrm{Out}}^{\prime }$s, which are the generated spin configurations through the input‐dependent and ‐independent processes, respectively, mentioned in Section 2.2. Though the amplitudes of the noises, which is the deviation from the stable spin direction, are mostly small and unnoticeable in the spin configuration images, they are clearly distinguished in the local correlation maps (right panels in *X*_Out_ and $X_{\mathrm{Out}}^{\prime }$ in Figure 2A). As *γ* increases, the noises in the local correlation maps are further reduced and the shapes of the magnetic skyrmions shown in the images become more plausible; the truncated or stretched shaped magnetic skyrmions frequently shown in the *X*_Out_ and $X_{\mathrm{Out}}^{\prime }$ of Figure 3A i disappear in Figure 3A iii.

To quantitatively analyze the local noise reduction, we investigate the distributions of *θ* (the angle between the spin and the effective field) as shown in Figure 3B. As *γ* increases, the *θ* distributions from *X*_Out_ and $X_{\mathrm{Out}}^{\prime }$ sharpen as the peaks shift toward zero. The decrease in *θ*, i.e., the spins aligning along the effective field, with increasing of the *L*_H_ term clearly demonstrates the energetic stability of the E‐VAE generated states.

### 3.4 Manipulated Latent Space

**Figure 4:**
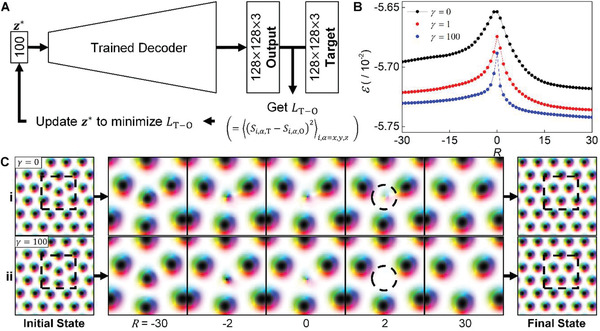
Interpolation results between the skyrmion lattice states with and without an interstitial defect. A) A schematic diagram showing the process to search the latent vectors which can be decoded to the specific target spin configurations. *L*_T − O_ is a mean squared error between an output which is decoded from z* and a target spin configuration. B) A graph for the energy density values, *ε*, calculated from the interpolated spin configurations between the skyrmion lattice states with and without an interstitial defect. *R* represents the interpolation steps away from the peaks of the energy curves. See Note S1 and Figure S2 in the Supporting Information for the exact definition of *R*. C) The visualized interpolation results from the initial state to the final state for the i) *γ*  =  0 and ii) *γ*  =  100 cases. The interpolation results (*R* = −30~30) are shown in a zoomed‐in (region within the dashed squares in the initial and final state) image to better emphasize the difference between (i) and (ii).

E‐VAE generates spin configuration from a randomly sampled latent vector *z*. In other words, the effects from *L*_H_ manipulate the structure of the latent space to generate energetically stable spin configurations. To investigate how $L_{H}$ affects the latent space, we interpolate between two latent vectors that can be decoded to two distinct spin configurations. One is a perfectly ordered magnetic skyrmion lattice, and the other is a magnetic skyrmion lattice with an interstitial defect as shown in **Figure** **4**. We use an inverse mapping method ^[^ ^39^ ^]^ to find the latent vectors decoded to the specific spin configurations. The schematics are shown in Figure 4A and the details are given in the Experimental Section. Figure 4B shows the energy values of the interpolated spin configurations between the two states. Since these spin configurations are similar except for only a single defect point, the energy differences between the two are small; yet, there is a peak indicating an energy barrier along the transition caused by the topological structure change. *R* in Figure 4B represents the number of interpolation steps from the peaks of interpolated energy curves and the positive and negative values of *R* represent the interpolated states closer to the final state (a perfectly aligned skyrmion lattice state) and the initial state (a skyrmion lattice state with an interstitial defect), respectively. Detailed information on the interpolation process and definition of *R* are given in Note 1 and Figure S2 in the Supporting Information. The peaks of interpolated energy curves sharpen as *γ* increases. To better visualize this, in Figure 4C, we show the spin configurations decoded from the *z*s for the *γ*  =  0 and *γ*  = 10^2^ cases. When *R*  =  2, the interstitial defect shown in the case of *γ*  =  0 is disappearing, yet still remains; for *γ*  = 10^2^ , the interstitial defect is already completely vanished. This clearly demonstrates that introducing $L_{H}$ results in the deformation of the latent space, leading to the sharpening of the energy barrier and the suppression of the generation of unstable states. In Figure 4B, note that introducing the Hamiltonian loss lowers the energy in the plateau region for each spin state, in addition to the sharpening of the energy barrier. This owes to the fact that the spins better align along the effective fields by a stronger *L*_H_ term, as discussed in Section 3.3.

### 3.5 Collapsed E‐VAE

**Figure 5:**
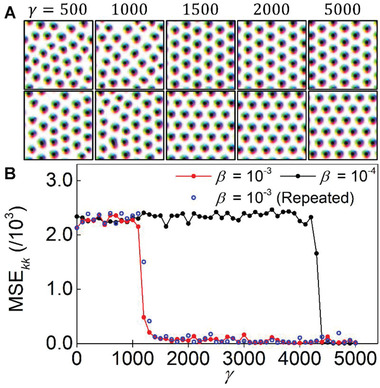
Collapsed E‐VAE. A) $X_{\mathrm{Out}}^{\prime }$s generated from the E‐VAE models trained under the *γ*  =  500, 1000, 1500, 2000, and 5000 with *β*  = 10^−3^ conditions. The first and second rows in (A) represent the training results performed under identical conditions. B) Mean squared error values calculated between the Fourier transformed spin configurations, MSE_*kk*_, generated from each trained E‐VAE model. The red and blue data points are obtained from two independently performed training cases under identical conditions.

As discussed in Section 3.2, there is a critical transition when the *γ* exceeds a critical value. Interestingly, above the critical value *γ*, the energy of the generated spin configurations suddenly drops to a low constant value. To better visualize the transition, we show the spin configuration change at *γ* values near the transition (**Figure** **5A**).

After the transition, the trained E‐VAE no longer generates various metastable states and only produces one magnetic state which is, in fact, the ground state of the system. We speculate that this phenomenon of the E‐VAE is similar to the aforementioned PC, except that it is induced by the *L*_H_ term, not the *L*_KL_ term. When *γ* exceeds the critical value, the *L*_H_ term dominates the total loss minimization process. In this case, the structure of the trained latent space is collapsed and changed over to generate a completely different spin configuration in the lowest possible energy states that can be inferred from the training process. We repeat the training process with the same conditions as shown in the second row of Figure 5A and find that each training case can be collapsed to generate a skyrmion lattice with a different orientation but with the same lowest energy. In other words, one can notice that the two generated spin configurations from the collapsed E‐VAEs are translated from a single perfectly ordered skyrmion lattice state.

In order to secure the quantitative evidence for the collapsing behavior of E‐VAE, we investigate the similarities between the generated spin configurations for each of the trained E‐VAE cases. For this, we compare the similarity in the reciprocal space (*k*‐space) by calculating a mean squared error (MSE) among the spin configurations, a method similar to that proposed in a previous study.^[^ ^40^ ^]^ We generate 100 $X_{\mathrm{Out}}^{{}^{\prime }}$s for each trained E‐VAE model. The out‐of‐plane component of these $X_{\mathrm{Out}}^{{}^{\prime }}$s, *S_z_* maps, are transformed into *k*‐space representations through a fast Fourier transformation, then the MSE values between all pairs in 100 transformed spin configurations are calculated. Each point shown in Figure 5B indicates the averaged MSE for each model trained using the given *γ* values. The higher values indicate that the trained models can still generate various spin configurations, while zero indicates that only one physical state is generated. This comparison in the reciprocal space confirms that the E‐VAEs are indeed collapsed to generate a single state that is the lowest energy state, as shown in Figure 5B. The critical value of *γ* required for the collapse depends on *β*. Yet, even for small *β*, eventually all cases collapse to the ground state when *γ* increases (Figure 5B).

This unique and surprising feature of the collapsed E‐VAE can be widely applied to search unknown ground states in various systems and conditions. To confirm the feasibility, we adopted the collapsing process of E‐VAE to search the ground states of skyrmion configurations in geometrically confined magnetic systems.^[^ ^41^, ^42^, ^43^ ^]^ Experimentally, skyrmion systems are frequently fabricated into non‐periodic square or wire shaped devices for skyrmion transport and dynamics studies. Here, let us consider a skyrmion system with a square boundary. In this case, the ground states of the skyrmion configuration are not intuitively predictable since the skyrmion lattice has a triangular lattice structure with sixfold symmetry (Figure 5A); such a structure cannot be fitted within the square boundary. We find that our E‐VAE can generate the lowest energy states for various boundary conditions (see Note S2 in the Supporting Information for the detailed process and results).

## 4 Conclusion

We devised a generative machine learning model, E‐VAE, which can generate various metastable states that are also energetically stable. This was done by including the Hamiltonian loss term to the standard VAE model. We found that the Hamiltonian loss reduces local noises, thereby naturally removing anomalies in the output. The Hamiltonian loss manipulates the latent space and makes energetically stable states more frequently sampled and thus suppresses improbable high energy structures in the generated spin configurations.

We also discovered the existence of a critical transition in the E‐VAE: when the strength of the Hamiltonian loss exceeds a critical value, the trained model loses the capability to generate various metastable spin configurations and only generates one magnetic state that is the ground state of the system. This collapsing behavior of E‐VAE induced by *L*_H_ suggests that our method has the potential to be utilized in various scientific research, specifically to explore uncharted ground states of various systems.

## 5 Experimental Section

##### Dataset Generation

39 300 spin configurations were generated using a simulated annealing process implemented by the Monte Carlo method with the magnetic Hamiltonian in Equation (1). During the annealing process, the temperature of the system decreased from above the Curie temperature to zero temperature to obtain various metastable spin states. Since the spontaneous symmetry breaking process was involved in the formation of magnetic domains, all generated magnetic states had different spin configurations with each other; it was highly unlikely that the total dataset contains redundant data. The total dataset was divided into three sub‐datasets to train the network structure (30 000 data for training dataset), to monitor the training process (5000 data for validation dataset), and to evaluate the performances of the trained network (4300 data for test dataset), respectively.

##### Detailed Network Structures

In the Encoder network structure shown in Figure 1, four convolutional neural network (CNN) ^[^ ^44^ ^]^ layers with 6, 12, 24, and 48 filters were used to extract the features of input data. The lateral sizes of all filters were identical (5  ×  5). Since the spin configuration dataset was generated under a periodic boundary condition, a periodic padding process was used before all CNN layers to implement identical conditions. Batch normalization layer,^[^ ^45^ ^]^ Leaky‐ReLU activation,^[^ ^46^ ^]^ and max‐pooling layer with 2  ×  2 pooling size were attached, in this order, after all the CNN layers in the Encoder structure. The extracted features from CNNs were fed to two dense neural network (DNN) layers containing 3072 and 512 neurons, respectively. Batch normalization layer and Leaky‐ReLU activation were attached only after the first DNN layer, and there was no activation process in the second DNN layer.

The Decoder network structure was composed of two DNN layers (512 and 3072 neurons for each), four upsampling processes (2  ×  2 upsampling size) with CNN layers, the last CNN layer without activation function, and the L2‐normalization process. The CNN layers in the upsampling processes had 24, 12, 6, and 3 filters for each, and three filters were in the last CNN layer. The lateral sizes of the filters in all CNN layers were 5  ×  5 identical to those of the Encoder. Also, a periodic padding process was used before all CNN layers in the Decoder, and Batch normalization layer and Leaky‐ReLU activation were attached after all CNN and DNN layers except the last CNN layer. As mentioned in the “Dataset and network structure” section, L2‐normalization process was added as the last part of the Decoder structure to make all spins in the output to be unit vectors. Adam optimizer was employed to minimize the losses in this study. The learning rate, *β*_1_, and *β*_2_ which were the hyper‐parameters of the Adam optimizer are fixed at 0.001, 0.9, and 0.999, respectively.

##### Sequential Training Process

Through a previous study ^[^ ^37^ ^]^ which proposed the *β*‐VAE model, it was known that a well‐trained VAE can be obtained by gradually increasing the *β* value in the training process. Hence, a standard VAE model was trained while increasing *β* value from 10^−6^ to 1. Specifically, the VAE was initially trained for 100 training epochs under the condition of *β*  = 10^−6^ , after which, the training process continued with an increased *β* value for another 100 training epochs. This sequential training process was continued until *β* reached 1.

In the training process of E‐VAE, a similar method was also used to control the *γ* value. Initially, two standard VAE models which were trained under the conditions of *β*  = 10^−3^ and 10^−4^ were employed as the initial models of E‐VAE training process. Then, these initial models were trained while sequentially increasing the *γ* value from 10^−4^ to around 10^6^ as shown in Figure 2B. During this process, the *γ* value was controlled per 100 training epochs while the *β* values were fixed. The batch size used in all training processes was 100.

##### Inverse Mapping Method

Figure 4A shows a schematic process to obtain the latent vector, z*, which can be decoded to a specific target spin configuration. First, two target spin configurations were created, which were the perfectly ordered skyrmion lattice and the skyrmion lattice with an interstitial defect, using a simple numerical technique. The energy of initial states which were composed of artificially arranged magnetic skyrmions was minimized by the greedy algorithm. Using the trained encoder which was separated from a trained E‐VAE model, the *μ*_*n*_ values were obtained from the target spin configurations. The z*, which were an updatable variable, was initialized using the *μ*_*n*_ values, and fed to the trained decoder to be decoded as an output spin configuration. The MSE was calculated between the output and target spin configurations, which was the *L*_T − O_ shown in Figure 4A, and let only the z* updated to minimize the *L*_T − O_. As this process progressed, the output spin configuration decoded from z* became similar to the target spin configuration. This process was run until the *L*_T − O_ value was minimized and saturated.

## Conflict of Interest

The authors declare no conflict of interest.

## Author Contributions

H.Y.K. created the design, developed the methodology, and performed network training. H.G.Y., S.M.P., and D.B.L. generated the total dataset and participated in the network training. J.W.C. and C.W. helped with the discussion of the results. H.Y.K and C.W. equally contributed to supervise the work progresses. All authors contributed to the writing of the paper.

## Supporting information

Supporting Information

## Data Availability

The data that support the findings of this study are available from the corresponding author upon reasonable request.
