# Supplementary material for: Magnetic State Generation using Hamiltonian Guided Variational Autoencoder with Spin Structure Stabilization
Source: Adv Sci (Weinh). 2021 Mar 24;8(11):2004795. doi: 10.1002/advs.202004795 (PMC8188203; doi:10.1002/advs.202004795)
Supplement: Supplementary file 1 — Supporting Information [file ADVS-8-2004795-s001.pdf]

© 2021 Wiley-VCH GmbH

ADVANCED SCIENCE
Open Access

Supporting Information

for *Adv. Sci.*, DOI: 10.1002/advs.202004795

Magnetic State Generation using Hamiltonian Guided Variational Autoencoder with Spin Structure Stabilization

Hee Young Kwon, Han Gyu Yoon, Sung Min Park, Doo Bong Lee, Jun Woo Choi, and Changyeon Won**

Supporting Information for

Magnetic state generation using Hamiltonian guided variational autoencoder with spin structure stabilization

H. Y. Kwon[1*], H. G. Yoon[2], S. M. Park[2], D. B. Lee[2], J. W. Choi[1], and C. Won[2*]

[1]*Center for Spintronics, Korea Institute of Science and Technology, Seoul 02792, South Korea*

[2]*Department of Physics, Kyung Hee University, Seoul 02447, South Korea*

The PDF file includes:

Figure S1, Figure S2, Figure S3

Note 1, Note 2

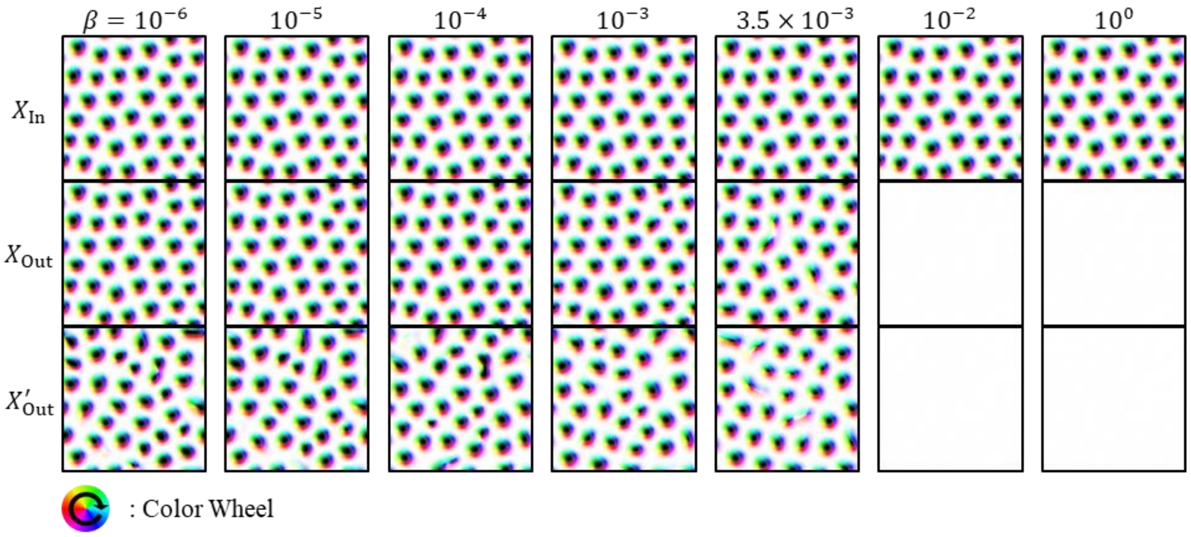

Figure S1. X_{Out}s and X'_{Out}s generated from standard VAE models. Each column shows the input and output spin configurations from each of standard VAE models trained with the β values written above. First row shows the input spin configuration which is fed to the trained model. To clearly compare what happens in the generated results, the input is set to be same for all beta cases. Second and Third rows show the output spin configurations, X_{Out}s and X'_{Out}s, respectively. The color wheel indicates the in-plane magnetization directions and black/white contrast indicates the out-of-plane magnetization directions.

Note 1.

The raw interpolation results between the latent vectors for a perfectly ordered skyrmion lattice and a skyrmion lattice including an interstitial defect are shown in Fig. S2A. From the initial state, an interstitial defect case, to the final state, a perfectly ordered skyrmion lattice case, 200 steps are used to interpolate between them. As mentioned in our manuscript, the interpolated energy curves shown in Fig. S2A have a single peak for each because, in order to evolve from the initial to the final state, there is only one topological energy barrier induced by a skyrmion that is used to create the interstitial defect.

One can notice that the x-axis of Fig. S2A is not indicating the definite positions in the latent space but indicating the interpolation steps. In fact, the positions of peaks in the latent space are not definite; i.e. if the E-VAE is trained again from the initial untrained network state, the single peak will probably be found at another location in the latent space. Therefore, we think that the absolute position in the latent space is not good coordinates to represent the interpolation results, and we use the interpolation steps instead.

In order to directly compare the peaks of different γ cases, we use a modulated coordinates, R, which indicates the number of interpolation steps from the peaks; the peaks shown in Fig. S2A are moved to 0 position of Fig. S2B, and the x-range is cropped from 30 steps before (negative) and after (positive) the peaks as shown in Fig. S2B.

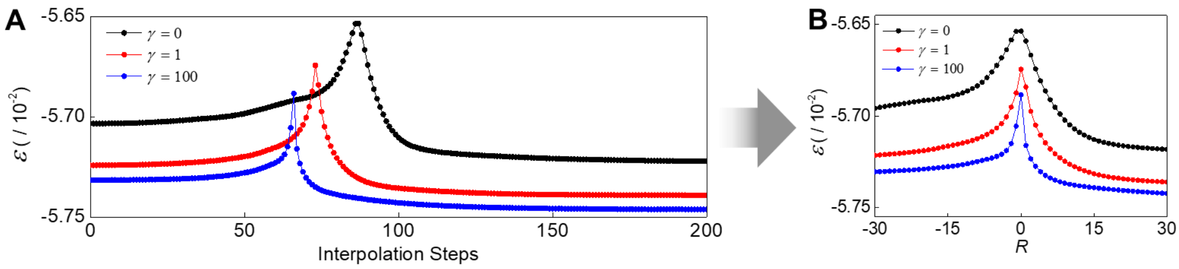

Figure S2. The energy curves of interpolated spin configurations between two specific states. Energy density, ε, are investigated for each γ case. A) The energy curves represented on the interpolation steps coordinates. 0 and 200 steps indicate the latent space position of initial and final state, respectively. B) The graph in Fig. 4B in our manuscript. In this graph, the interpolated energy curves are represented on the modulated coordinates, R, which is the interpolation steps from each of peaks.

Note 2.

To get the collapsed E-VAE models for each of systems with different boundary conditions, we used the sequential training process to increase the γ parameter from zero to 2500 under $\beta = 10^{-3}$ condition. (The collapsing condition of E-VAE when $\beta = 10^{-3}$ is around $\gamma = 1000$ as shown in Figure 5B) The training dataset which is used to train the VAE and E-VAE models discussed in our manuscript also used in this case, but the calculation process of Hamiltonian loss term is slightly changed to implement the geometrical confinements. We forced some regions of output spin configurations to have zero magnetization, and used the modified spin configurations to calculate our Hamiltonian loss term during the whole training process. For example, in the case shown in Figure S3B, the regions where are fixed to have zero magnetization are 6 grid sites of all leftmost, rightmost, topmost, and bottommost regions of output spin configurations. Through this techniques, we could implement the boundaries of the confined systems, and could get the proper ground states for all cases considered in Figure S3.

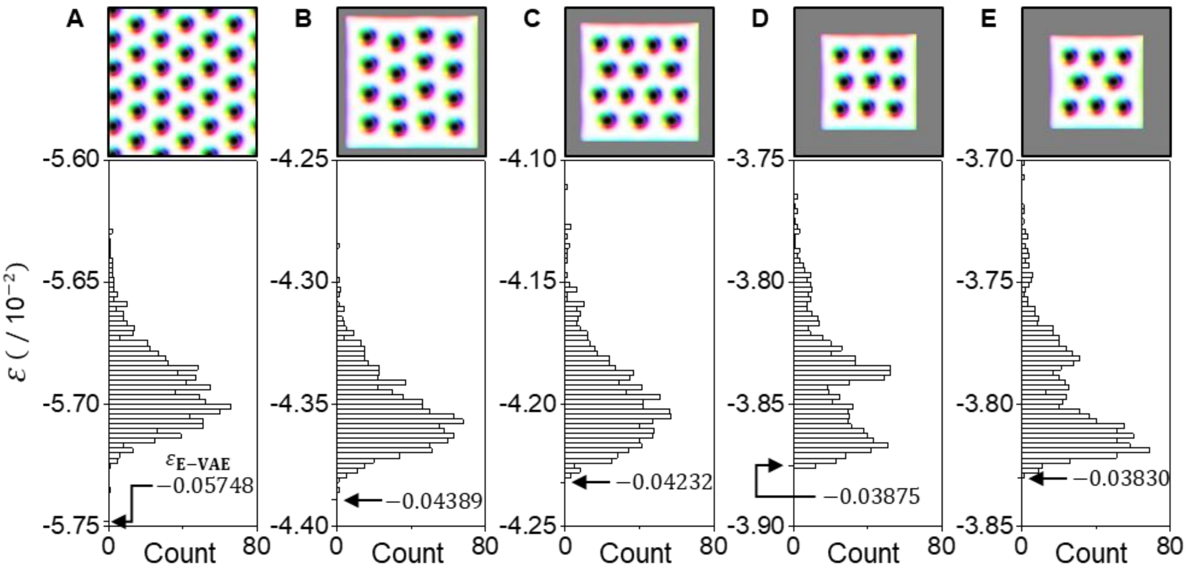

Figure S3. The lowest energy states generated by E-VAE for the systems with various boundary conditions. The spin configurations from collapsed E-VAE and energy distributions of 1000 spin configurations generated by a simulated annealing method for each of (A) a periodic system, (B-E) the systems with non-magnetic boundaries of (B) 6 grids, (C) 12 grids, (D) 22 grids, and (E) 23 grids, respectively. The energies of each spin configuration, $\varepsilon_{\mathrm{E-VAE}}$, are indicated by black arrows in each of histogram graphs.
